# AFibNet: an implementation of atrial fibrillation detection with convolutional neural network

**DOI:** 10.1186/s12911-021-01571-1

**Published:** 2021-07-14

**Authors:** Bambang Tutuko, Siti Nurmaini, Alexander Edo Tondas, Muhammad Naufal Rachmatullah, Annisa Darmawahyuni, Ria Esafri, Firdaus Firdaus, Ade Iriani Sapitri

**Affiliations:** 1 Intelligent System Research Group, Faculty of Computer Science, Universitas Sriwijaya, Palembang, 30139 Indonesia; 2 Department of Cardiology and Vascular Medicine, Dr. Mohammad Hoesin Hospital, Palembang, Indonesia

**Keywords:** Cloud deep learning, 1D-convolutional neural network, Atrial fibrillation

## Abstract

**Background:**

Generalization model capacity of deep learning (DL) approach for atrial fibrillation (AF) detection remains lacking. It can be seen from previous researches, the DL model formation used only a single frequency sampling of the specific device. Besides, each electrocardiogram (ECG) acquisition dataset produces a different length and sampling frequency to ensure sufficient precision of the R–R intervals to determine the heart rate variability (HRV). An accurate HRV is the gold standard for predicting the AF condition; therefore, a current challenge is to determine whether a DL approach can be used to analyze raw ECG data in a broad range of devices. This paper demonstrates powerful results for end-to-end implementation of AF detection based on a convolutional neural network (AFibNet). The method used a single learning system without considering the variety of signal lengths and frequency samplings. For implementation, the AFibNet is processed with a computational cloud-based DL approach. This study utilized a one-dimension convolutional neural networks (1D-CNNs) model for 11,842 subjects. It was trained and validated with 8232 records based on three datasets and tested with 3610 records based on eight datasets. The predicted results, when compared with the diagnosis results indicated by human practitioners, showed a 99.80% accuracy, sensitivity, and specificity.

**Result:**

Meanwhile, when tested using unseen data, the AF detection reaches 98.94% accuracy, 98.97% sensitivity, and 98.97% specificity at a sample period of 0.02 seconds using the DL Cloud System. To improve the confidence of the AFibNet model, it also validated with 18 arrhythmias condition defined as Non-AF-class. Thus, the data is increased from 11,842 to 26,349 instances for three-class, i.e., Normal sinus (N), AF and Non-AF. The result found 96.36% accuracy, 93.65% sensitivity, and 96.92% specificity.

**Conclusion:**

These findings demonstrate that the proposed approach can use unknown data to derive feature maps and reliably detect the AF periods. We have found that our cloud-DL system is suitable for practical deployment

## Introduction

A single pulse of an electrocardiogram (ECG) signal consists of the morphology, heart rate, regularity, wave segments, relative amplitudes, timing intervals, and normalized energy in a beat or a rhythm [1]. ECG is a popular non-invasive tool used to classify healthy and unhealthy cardiac activity based on a time series signal [2, 3]. An estimated 300 million or more ECGs are recorded worldwide every year [2], representing a tremendous amount of data for cardiologists to analyze.

One electrophysiologic disturbance within the atria that can be observed by ECG is termed atrial fibrillation (AF) [2]. AF is the most prevalent severe abnormal heart rhythm associated with a fast heart rate. It refers to an abnormal, rapid, and non-synchronized muscle fiber contraction with complex patophysiology [4–7], and is recognized as an independent risk factor for stroke, with important clinical and economic consequences. Diagnosing the symptoms is important before treatment of this severe disease; however, existing commercial ECG devices for AF detection methods still show actual misdiagnosis rates. This is largely due to the lack of generalizability caused by tuning only for specific medical devices [8].

Continuous or real-time monitoring of an ECG may help distinguish heart abnormalities. All ambulatory 12-lead ECG systems are designed to ensure reliable AF detection. In environments such as primary care centers and emergency units, where no experts are available to examine and interpret ECG tracings, these ECG devices are commonly used. Unfortunately, these devices are pricey, time-consuming, challenging to use, and require long-term exposure for AF measurements [9]. An automatic and accurate interpretation is critical in low and middle-income countries, and could potentially prevent 75% of cardiovascular disease deaths [9], as those populations often do not have access to cardiologists with full expertise in ECG diagnosis. In fact, medical professionals in those environments typically have limited diagnostic expertise in interpreting 12-lead ECGs [9, 10]. Therefore, a simple AF detection that does not require hospital visits and is publicly accessible is required for better diagnosis.

The use of single-lead ECG with short-term detection is currently prevalent in daily applications because the device is simple, low cost, and easy to use [11]. Regardless, AF detection using short-term signal detection can be missed in many cases due to the lack of data standardization collection, the processing procedures used, and the inconsistent reporting of technological factors, such as frequency sampling [12, 13]. Several short-term ECG instruments have variable signal quality, frequencies, and lengths for detecting AF episodes. Data may also need to be sampled at a different target frequency, when dealing with multiple instruments that are sampled at various frequencies. However, choosing data sampled at a certain frequency will influence the generalization potential and complexity of the model [13]. Therefore, the chosen approach must be robust without decreasing effectiveness of the device to detect AF [13]. Hence, a basic approach for enhancing short-term AF identification with acceptable results is desirable.

Many of the computer-aided ECG signals proposed for AF detection over the past 50 years are based on machine learning (ML) [14] and have been used in commercial ECG medical devices [15]. Two significant bottlenecks that still hinder early auto-detection are the energy limitations of the continuous monitoring equipment and the lack of efficient ML-based models for AF prediction. In addition, conventional ML requires a separate technique of feature engineering that can be computationally expensive. The desired ML-based solution for automatic AF diagnosis therefore requires high accuracy but it also needs to be computationally efficient [16].

Recently, deep learning (DL) methods have shown great potential in the healthcare and medical areas [17, 18]. Specifically, some pioneering work has shown success in using DL methods for AF detection [19–21]. DL models can be trained to perform beat and rhythm detection/classification using ECG data collections but, unfortunately, the use of DL for AF detection remains essentially unexplored [22]. One DL approach is to use convolutional neural networks (CNNs) architecture with feature engineering embedded into the learning structure. Actually, the CNNs is a type of DL that excels in processing 2D data, such as images. However, by considering signals as 1-dimensional (1D) data, studies have shown promising results using convolutions for signal processing [20, 23–25]. Operations on a 1D-CNNs are only scalar multiplication, not matrix multiplication like two-dimensional (2D) CNNs. Therefore, the computational costs on the 1D-CNNs are about the same as the traditional machine (ML) methods. However, the traditional ML needs a feature engineering step that requires domain knowledge. Due to the feature engineering process, the inference pipeline of the ML algorithms becomes longer compared to DL algorithms [13]. Moreover, 1D-CNNs show superiority in AF signal processing and have outperformed both recurrent neural networks (RNNs) and deep neural networks (DNNs) [13].

Previous studies have shown that 1D-CNNs can successfully provide fast and accurate classification of long-term ECG records. They can analyze the morphological characteristics and learn the slit variation of an input signal during a short-term ECG [13]. The 1D-CNNs model is developed for patient-specific ECG classification [26]. A nine-layer CNNs model for classifying five types of heartbeats from initial signals used an augmentation technique and had a precision of 94.03% [27]. A 1D-CNNs model consisting of 33 convolutional layers based on a massive ECG dataset of 91,232 records from 53,549 patients was able to identify 12 rhythm categories [28]. A generic CNNs has been presented for patient-specific ECG classification [29]. The use of modified U-net architecture has been suggested to diagnose beatwise arrhythmia [30]. A 31-layer 1D residual CNNs model was developed to identify five different types of heartbeats [31]. A customized CNN model has been recommended to classify patient-specific heartbeats using 44 records [32]. A CNNs model has been applied for classification of 17 cardiac arrhythmias using long-duration ECG signals [33]. An end-to-end deep learning model has been proposed to classify 15 ECG classes [34]. However, despite this extensive study of the 1D-CNNs algorithm for classification/detection of ECG wave signals, the robustness of this algorithm remains an important issue and these methods are still far removed from practical applications [35].

Computerized ECG signal interpretation plays a critical role in the clinical workflow. Digital ECG data are readily accessible and the DL algorithmic model offers an opportunity to greatly increase the precision and scalability of automated ECG analysis [28]. A comprehensive evaluation of an end-to-end DL approach for AF ECG analysis across a wide variety of diagnostic devices has not been previously reported. None of the current models have been deployed to provide publicly available ECG AF detection services. Therefore, the aim of the present study was to propose a cloud-based 1D-CNNs approach that can be used to enhance AF detection based on CNNs by connecting it to the internet (AFibNet). This approach can provide easy and early detection of a potential AF anytime and anywhere.

This study make the following novel contributions are:

- Implementing an end-to-end of AF detection in a broad range of distinct ECG devices
- Develoving a generalization model for 1D-CNNs into a single learning system named AFibNet
- Implementing the proposed AFibNet model in a cloud deep learning system with 11,842 subjects for two-class classification (N and AF) with inter-patient mechanism and 26,349 subjects for three-class classification (N, AF, and Non-AF) with intra-patient mechanism
- Validating of the robustness of the proposes model through a cloud system in preparation for publicly available ECG AF detection services.

The rest of this paper is organized as follows: “Methods” section explains the method, and “Result and discussion” section presents the result and discussion. Finally, the conclusions are presented in “Conclusion” section.

## Methods

**Fig. 1:**
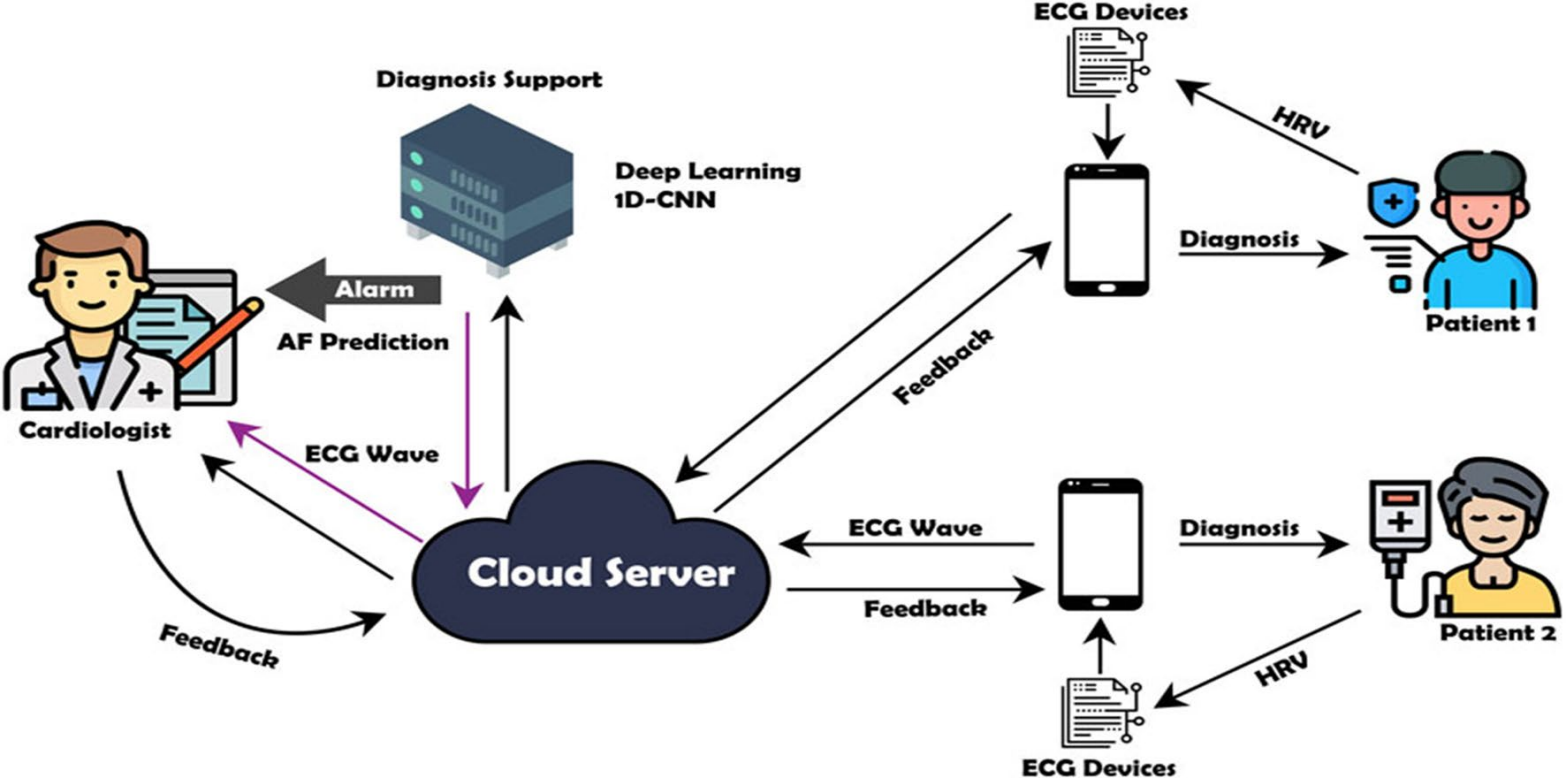
Flow diagram of deep learning model for AF diagnosis system

Currently, health care information technology uses a cloud service to develop a system that combines medical devices and applications [24, 36, 37]. The use of these technologies connects patients to their physicians and facilitates the sharing of medical data over a safe network, thereby eliminating needless hospital visits and lessening the burden on the health care system [28]. The patients can measure their own heart conditions and the measurement results of ECG signal recording are delivered to a central storage location for centralized decision-making. These measurement data are usually physiological signals in the cardiac ECG signal domain, such as beat, rhythm, and HRV [38]. The patients use short-term ECG devices and transmit the HRV signals to a mobile device for relay to the cloud server. Figure 1 shows a framework for collecting and analyzing ECG device data from a cloud server. Once the ECG data are collected, they are transferred to the mobile terminal via Bluetooth and displayed in real time, then transmitted to the cloud through WIFI or 4G [39]. The DL architecture then validates and analyzes the incoming HRV signals in real time. If the model detects AF in the signals, a cardiologist is informed. The cardiologist can then review the suspicious HRV trace as a beat or rhythm and reach a diagnosis. The diagnosis can later be communicated to the patient in a simple scheme.

This work proposes only the DL-based cloud system with the 1D-CNNs model in the main processing system for AF diagnosis support. The cloud-based DL is important for the AF diagnosis as it provides an accurate medical interpretation system. To ensure that the implementation works properly in all stages, all parts of the DL-based cloud system design are presented as follows.

### Dataset

In this study, nine public ECG databases are utilized: the MIT-BIH Atrial Fibrillation [40], the 2017 PhysioNet/CinC Challenge [41], the China Physiological Signal Challenge 2018 [42], ECG Long Term AF [43], Paroxysmal AF [41], AF Termination Challenge [44], Fantasia [45], MIT-BIH Arrhythmia [46], and ECG recording from Chapman University and Shaoxing People’s Hospital [47], as well as two ECG signal recording databases collected from an Indonesian Hospital. Based on all these datasets, we separated three main processes: training, validating, and testing. In this process, all data sets differed in the lengths of signal recording and frequency sampling. All databases were collected from a short single-lead recording with different ECG devices. As a result a diverse length of signal recording was formed varying from 10 s to 25 h and frequency varying from 100 to 500 Hz.

In the experimental study to measure the generalization and robustness of the proposed model, we conducted two cases evaluation, the two-class classification (N and AF) and three-class classification (N, AF, Non-AF). For two-class case, three datasets (MIT-BIH Atrial Fibrillation, the 2017 PhysioNet/CinC Challenge, and the China Physiological Signal Challenge 2018 databases), was used for training and validating. The model then was tested using the other eight datasets. The summary and samples of the ECG dataset used in this study for two-class case are shown in Table 1. As we can seen on Table 1, the total subjects consist of 7409 training, 823 validation, and 3610 testing/unseen data. The total records for N and AF rhythm contained 7784 N and 4058 AF records.

**Table 1:** ECG Record description for two-class case (N and AF)

Dataset	Frequency sampling (Hz)	Class	Records	Training data	Validation data	Testing/unseen data
PhysioNet/CinC challenge 2017	300	N	5154			–
		AF	771			–
China physiological signal challenge 2018	500	N	918	7409	823	–
		AF	1098			–
MIT–BIH atrial fibrillation	250	AF	291			–
MIT–BIH arrhythmia	360	AF	6	–	–	–
ECG long term	128	AF	38	–	–	–
Paroxysmal AF	128	AF	48	–	–	–
AF termination challenge	128	AF	10	–	–	–
Fantasia	250	N	24	–	–	–
		N	1646	–	–	–
ECG recording from Chapman University and Shaoxing People’s Hospital	500	AF	1780	–	–	3610
Indonesian Hospital (ECG 1)	500	N	42	–	–	–
		AF	3	–	–	–
Indonesian Hospital (ECG 2)	400	AF	13	–	–	–
		Total		7409	823	3610

The three-class case, five datasets (MIT-BIH Atrial Fibrillation, The 2017 PhysioNet/CinC Challenge, The China Physiological Signal Challenge 2018, ECG recording from Chapman University and Shaoxing People’s Hospital, and ECG recording from an Indonesian Hospital) was used for training and validating. However, due to data limitation, the trained model was not tested on unseen data. Table  2 shows the data description of ECG signal from 18 arrhythmias condition as indicated Non-AF class. The Non-AF class consists of 18 arrhythmias condition. The summary of Non-AF can be presented in Table 2, which consist of 7898 N, 3940 AF, and 14511 Non-AF records.

**Table 2:** ECG record description for three-class case (N, AF, and Non-AF)

Dataset	Frequency sampling (Hz)	Conditions	Class	Records	Training data	Validation data
PhysioNet/CinC challenge 2017	300	N	N	5154		
		AF	AF	771		
		Others	Non-AF	2557		
		Noisy		46		
		Normal	N	918		
China physiological signal challenge 2018	500	AF (Atrial Fibrillation)	AF	1098		
		I-AVB (First-degree atrioventricular block)		704		
		LBBB (Left bundle branch block		207		
		RBBB (Right bundle branch block	Non-AF	1695		
		PAC (Premature atrial contraction)		574		
		PVC (Premature ventricular contraction)		653		
		STD (ST-segment elevated)		826		
		STE (ST-segment elevated		202	23,714	2635
MIT-BIH trial fibrillation	250	AF (Atrial Fibrillation)	AF	291		
ECG recording from Chapman University and Shaoxing People’s Hospital	500	SR (Sinus rhythm)	N	1826		
		AF (Atrial fibrillation)	AF	1780		
		SB (Sinus bradycardia)		3889		
		ST (Sinus tachycardia)		1568		
		AFL (atrial flutter)		445		
		SI (Sinus irregularity)		399		
		SVT (Supraventricular tachycardia	Non-AF	587		
		AT (Atrial tachycardia)		121		
		AVNRT (Atrioventricular node reentrant)		16		
		AVRT (Atrioventricular reentrant tachycardia)		8		
		SAAWR (Sinus atrium to atrial wandering rhythm)		7		
Indonesia Hospital (ECG 1)	500	Non-AF (other rhythms)	Non-AF	7		

The total ECG data from 11,842 subjects for two-class and 26,349 for three-class cases, can be described as follows:

- MIT-BIH atrial fibrillation
  This database has 23 public ECG recordings taken from AF patients every 10 h. The ECG signals were sampled at 250 Hz, with four types of rhythm annotations such as AF, atrial flutter, AV junctional rhythm, and all other rhythms. The analog ECG recordings were made at the Beth Israel Deaconess Medical Center using ambulatory ECG recorders with a typical recording bandwidth of approximately 0.1–40 Hz.
- The 2017 PhysioNet/CinC Challenge
  All ECG records were sampled at 250 Hz by a single lead with four types of rhythms such as N, AF, Non-AF, and Noisy. We selected the recordings from N, AF, and Non-AF rhythms. All ECG recordings were collected using the AliveCor device for 9 to 60 seconds.
- The China Physiological Signal Challenge 2018
  This database was collected from 11 hospitals sampled at 500 Hz, with ECG normal and abnormal types. All 12-lead ECG recordings lasted from 6 to 60 s and were taken from 3178 female and 3699 male patients. The present study used only single lead (Lead II) data which consist of N rhythm about 981 records, AF rhythm about 1098 records, and Non-AF rhythms about 4861 records, respectively.
- ECG Long Term AF
  This database has 84 long-term ECG recordings of subjects with paroxysmal or sustained AF. Each record was digitized at 128 Hz, and the durations vary but are typically 24–25 h. The 38 records indicated as AF termination rhythm were utilized in the present study. The original recordings were digitized and automatically annotated at Boston’s Beth Israel Deaconess Medical Center. Steven Swiryn and George Moody annotated the AF terminations.
- Paroxysmal AF
  This challenge database consists of 50 pairs of half-hour ECG recordings sampled at 128 Hz. The database consists of group A, who experienced Paroxysmal AF (PAF) rhythm and group N rhythm who did not have PAF. We have tested group A only, with a total of 48 records.
- MIT-BIH Arrhythmia
  The database was digitized at 360 samples per second and contains 48 half-hour excerpts of two-channel ambulatory ECG recordings. The database was obtained from 47 subjects studied by the BIH Arrhythmia Laboratory between 1975 and 1979. The database has two types: beats and rhythms. This study tested the AF rhythm type of the ECG recordings (records 201, 203, 210, 217, 219, and 221).
- AF Termination Challenge
  This database is divided into a learning set and two test sets. The learning set contains 30 AF rhythm records in total, with 10 records in each of three groups (N, S, and T). Each record was sampled at 128 Hz, and the segments were extracted from 20–24 h ECG recordings. The ECG recordings were created for use in the Computers in Cardiology Challenge 2004. Among the three groups, the present study used group T, in which the AF terminates immediately.
- Fantasia
  All ECG recordings of N rhythm subjects were digitized at 250 Hz. Each set includes the respiration belt data from 20 young (21–34 years old) and 20 elderly (68–86 years old) subjects. For unseen data testing, the present study used only 24 records randomly chosen from the young and elderly cohorts. The respiration signals were collected by 120 minutes of continuous supine resting while under continuous ECG.
- ECG recording from Chapman University and Shaoxing People’s Hospital
  This database includes a large number of individual subjects (more than 10,000) with 12-lead ECG signals sampled at a higher than usual sampling rate of 500 Hz. The database includes 11 heart rhythms and 56 types of cardiovascular conditions labelled by professional physicians. The ECG records were acquired over 10 seconds. The ECG recordings were collected from 10,646 patients, including 5956 males and 4690 females. In the present study, we utilized 1826 N rhythm, 1780 AF rhythm and 7040 Non-AF rhythms data selected from Lead II.
- ECG recording from an Indonesian Hospital
  The Indonesian Hospital dataset contained sampled at 500 Hz (ECG 1) and 400 Hz (ECG 2). For ECG 1, the database consist of N rhythm about 42 records, AF rhythm about 3 records and Non-AF rhythms about 7 records. In addition, 13 AF rhythm records for ECG 2. All ECG records were collected for 10 s. The ECG database was collected by clinicians from patients who use ambulatory ECG devices (February to June 2020).

**Fig. 2:**
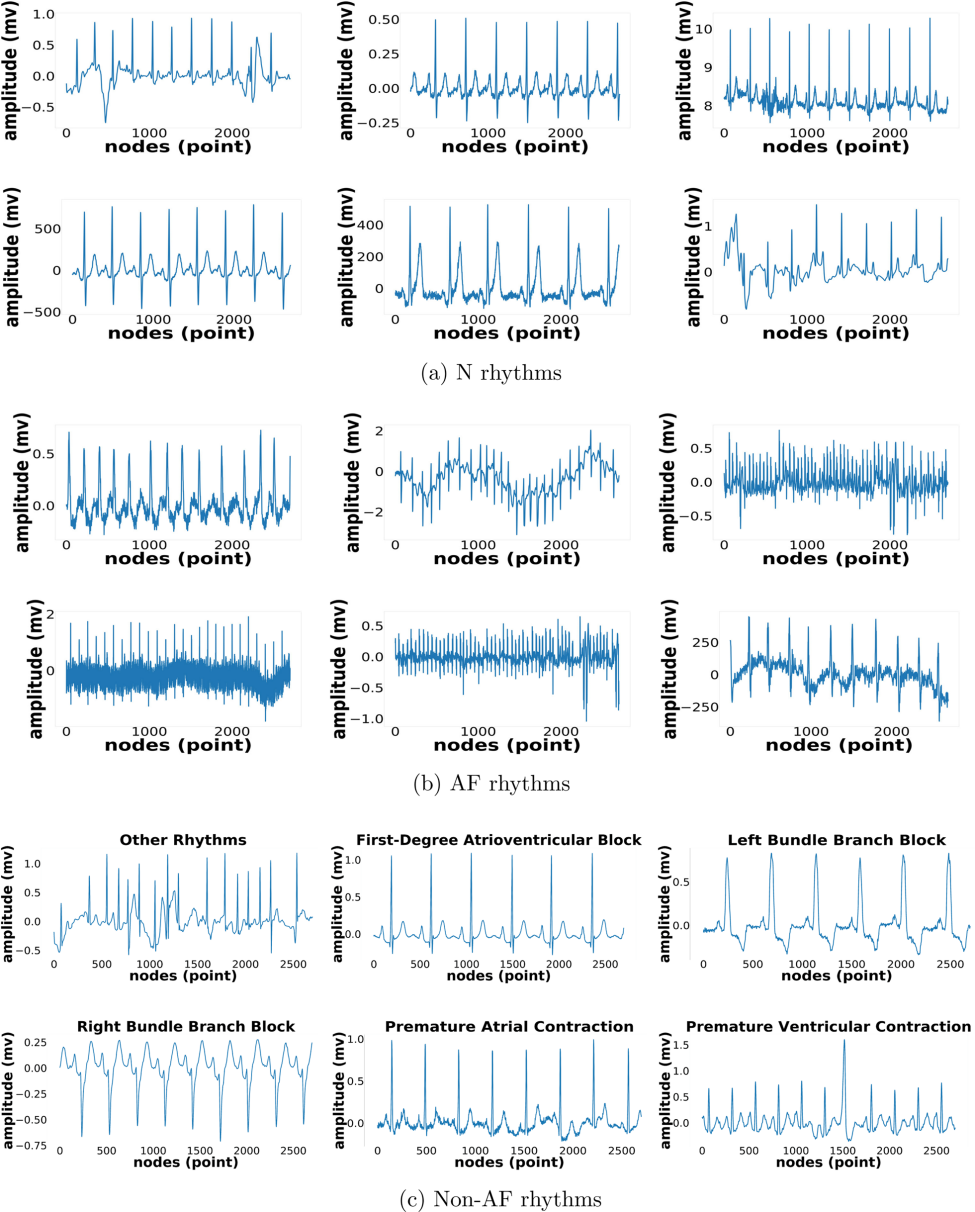
Sample of ECG raw data for (**a**) N, (**b**) AF, and (**c**) Non-AF rhythms from 11 total datasets with several devices, length of recording and frequency sampling

The samples of ECG signals of N, AF, and Non-AF rhythms are shown in Fig. 2. Figure 2a, b show the N and AF rhythm, and Fig. 2c presents other 18 arrhythmias rhythm. All samples of ECG raw data is taken from 11 datasets, which shown the difference of whole samples due to varying length of recording and frequency sampling.

### Implementation of atrial fibrillation detection

**Fig. 3:**
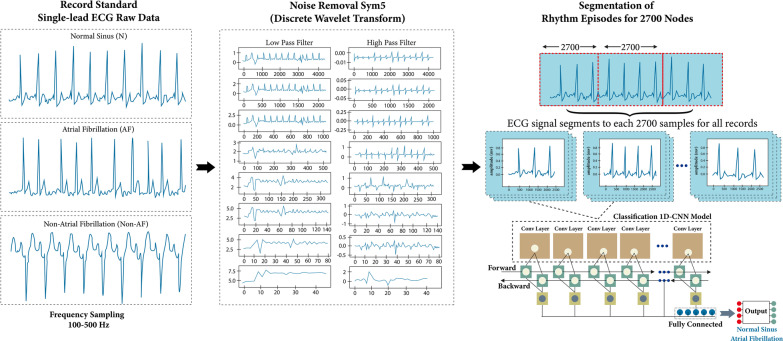
Proposed the AFibNet methodology

In this study, the proposed classifier for two-class and three-class cases are based on 13 convolution and 5 max-pooling layers of ID-CNNs architecture, which we have published in detail in a previous work [13]. However, in this study we improved the generalization and robustness of the proposed model by using input from several devices with different frequency samplings and lengths of the ECG signal waveform. In addition, it verified and tested in clinical settings with intra-patient and inter-patient scheme. Figure 3 shows the AFibNet methodology, which consisted of the following six main steps as follow;

- The large ECG signal data recording was used in this study, about 11,842 subjects for two-class classification (N and AF rhythms) and about 26,349 subjects for three-class classification (N, AF, and Non-AF rhythms) from 11 datasets for training, validating, and testing process. We use inter-patients mechanism in two-class classification case, where the training/validating dataset is difference with testing dataset. All data are taken from single-lead ECG standard recordings with varying lengths of signal and frequency sampling (100–500 Hz).
- ECG noise removal using discrete wavelet transform (DWT). This step decompose the ECG signals into the specific wavelet levels (8 levels) with Sym5 [13]. The signal frequency is divided by two in DWT because it passes through the high pass and low pass filters. Frequencies that pass through the high pass filter will enter the detail coefficient, while the low pass filter will enter the approximation coefficient;
- All ECG signals are segmented into 2700 samples for one episode. If the total nodes are less than 2700 nodes, we add zero-padding technique that consists of extending a signal with zeros. An AF rhythm may contract at up to 600 beats per minute (bpm), thereby creating a high irregularity of R–R intervals and a sudden disappearance of regularly occurring P-waves [40, 48]. Therefore, at least three to four heartbeats are needed to represent the AF episodes [40]. To assess the R–R interval in all ECG records, we have considered the minimum and maximum lengths for ECG signal segmentation based on the training datasets mentioned earlier. The ECG segmentation of 2700 nodes contained at least two R–R intervals or three beats with different frequency samplings (250 Hz, 300 Hz, and 500 Hz) in all records. In addition, with a minimum frequency sampling of 128 Hz for the testing set, the 2700 nodes segmentation could present more than two R–R intervals. Hence, 2700 nodes for ECG segmentation were selected as the best ECG episodes.
- Two features are generated from ECG signal irregularly irregular of heart rhythm and the maksimum of amplitude as R-peak in one episodes of ECG signal, and it learn episode by episode. The feature is represented by ECG signal amplitude along 2700 nodes. The most important process of the 1D-CNNs method is that the common cause of AF is modeled by a series of filters in the convolution layer and sub sampling in the maxpooling layer. The feature output is used to synthesize the corresponding potential abnormal and normal rhythms. The feature reduce from 2700 nodes in layer-1 becomes 78 nodes in layer-13 with maxpooling-5, and the selected feature that use as input in fully connected layer to classify the normal and AF feature.
- Each ECG signal episodes of 2700 nodes was trained using the 1D-CNNs classifier model was proposed by Nurmaini et al. [13]. The structure model has 13 hidden layers with an activation function rectified linear unit (ReLU) in the hidden layers and tanh-sigmoid in the output layers [13]. The hyperparameters utilize a 0.0001 learning rate, 16 batch size, and 100 epochs. The training process for AF detection was fully supervised. It back-propagated the gradients from the fully connected layer through to the convolutional layers. As a loss function, we minimized the binary cross-entropy to optimize the model parameters, and we utilized the gradient descent with the Adam update rule.
- The 1D-CNNs model was proposed with several hardware platforms and software frameworks using both local (on-device) and remote (network-side server) computation (refer to Fig. 1). The DL-based cloud system is designed to process the AF detection and to ensure that the proposed model works properly in real applications. Therefore, the computational complexity is deeply analyzed. Three parameters of the computational complexity as a cloud performances, namely processing time, throughput, and testing time, are validated using a computer with and without GPU. The memory consumption is one of the parameters to be considered, based on the selected classifier model. Each process in the convolution layer that is fully connected can be counted as memory consumption in our model and can be calculated from output shape from each layer parameter in the CNNs architecture.

### Feature learning in 1D-CNNs

1D-CNNs architecture has two distinct layer types, followed by CNNs-layer and then fully-connected (FC) layer. The feature learning was processed in CNNs-layer by using convolution and sub-sampling (pooling) process. The specific function of the two layers is for reducing the complexity and dimension of the ECG feature. In this study, we generate one by one episode (2700 nodes) as a feature along with ECG signal recording. The amplitude is represented by each node from node 1 to 2700. The CNNs-layers process and learn to extract features (feature learning) of the raw 1D data, which are used in the classification task performed by the FC-layers. As a result, both feature extraction and classification operations are integrated into one process that can be streamlined to improve the performance of the classification. All feature learning process is explained detail in Table 3.

In the convolution process, several unique features are generating. For example, in convolution layer 1, was feed with 2700 nodes of the ECG signal. This layer has 64 kernels with a size of 3 $\times$ 1 and a stride of 1. This striding process is carried out along the ECG signal so that it can produce 3 features at the beginning, middle, and end of the signal episode. Then, the result of the convolution process is continued to the max-pooling layer. The pooling layer aims to summarize the features resulting from the convolution process so that it not only reduces the computation load but also can strengthen the model against variations in the input signal.

**Table 3:** Feature learning interpretation

Layer	Input nodes	Filter number	Kernel size/pool size	Output nodes	Feature interpretation
Input	2700, 1	–		–	ECG amplitude for one episode
Convolution 1	2700, 1	64	3 $\times$ 1, stride 1	2698 $\times$ 64	64 feature map
Convolution 2	2698 $\times$ 64	64	3 $\times$ 1, stride 1	2696 $\times$ 64	64 feature map
Max pooling 1	2696 $\times$ 64	–	2 $\times$ 1, stride 2	1348 $\times$ 64	Feature reduction (1348 nodes for one episode)
Convolution 3	1348 $\times$ 64	128	3 $\times$ 1, stride 1	1346 $\times$ 128	128 feature map
Convolution 4	1346 $\times$ 128	128	3 $\times$ 1, stride 1	1344 $\times$ 128	128 feature map
Max pooling 2	1344 $\times$ 128	–	2 $\times$ 1, stride 2	672 $\times$ 128	Feature reduction (672 nodes for one episode)
Convolution 5	672 $\times$ 128	256	3 $\times$ 1, stride 1	670 $\times$ 256	256 feature map
Convolution 6	670 $\times$ 256	256	3 $\times$ 1, stride 1	668 $\times$ 256	256 feature map
Convolution 7	668 $\times$ 256	256	3 $\times$ 1, stride 1	666 $\times$ 256	256 feature map
Max pooling 3	666 $\times$ 256	–	2 $\times$ 1, stride 2	333 $\times$ 256	Feature reduction (672 nodes for one episode)
Convolution 8	333 $\times$ 256	512	3 $\times$ 1, stride 1	331 $\times$ 512	512 feature map
Convolution 9	331 $\times$ 512	512	3 $\times$ 1, stride 1	329 $\times$ 512	512 feature map
Convolution 10	329 $\times$ 512	512	3 $\times$ 1, stride 1	327 $\times$ 512	512 feature map
Max pooling 4	327 $\times$ 512	–	2 $\times$ 1, stride 2	163 $\times$ 512	Feature reduction (163 nodes for one episode)
Convolution 11	163 $\times$ 512	512	3 $\times$ 1, stride 1	161 $\times$ 512	512 feature map
Convolution 12	161 $\times$ 512	512	3 $\times$ 1, stride 1	159 $\times$ 512	512 feature map
Convolution 13	159 $\times$ 512	512	3 $\times$ 1, stride 1	157 $\times$ 512	512 feature map
Max pooling 5	157 $\times$ 512	-	2 $\times$ 1, stride 2	78 $\times$ 512	Feature reduction (78 nodes for one episode)
Flatten	39,936	–	–	–	Dot product between 78 nodes and 512 feature map
Dense	–	–	–	1000	Weight params
Dense	–	–	–	1000	Weight params
Output	–	–	–	1	Class

## Result and discussion

### 1D-CNNs classifier performances

As stated before, this study generated two-cases model: two and three-class case of AF classification. For each case, can be described as follows.

#### Test case 1: N and AF classification (two-class)

Based on the data distribution, 6072 and 2160 records for N and AF signals were used in the training and validation process. All ECG data (about 8232 records), after segmentation at 2700 for each record, produced 54,038 rhythm episodes. In order to avoid overfitting before the 1D-CNNs model was generated, a k-fold (k $=$ 10) validation technique was used to tune the class weight with the resampling procedure for the total data bias [13]; the performances reported in the results are the average scores. All data are split as 90% for the training process and the remainder for the validation process and are resampled tenfold again. Table 4 shows the data distribution of the N and AF condition data in each fold’s training and validation set.

**Table 4:** Data segementation with a tenfold scheme for a combination of three datasets (MIT-BIH Atrial Fibrillation, the 2017 PhysioNet/CinC Challenge, the China Physiological Signal Challenge 2018 databases)

Fold	Training data		Validation data		Total
	N	AF	N	AF	
1	16,485	32,149	1790	3614	54,038
2	16,391	32,243	1884	3520	54,038
3	16,424	32,210	1851	3553	54,038
4	16,402	32,232	1873	3531	54,038
5	16,469	32,165	1806	3598	54,038
6	16,520	32,114	1755	3649	54,038
7	16,476	32,158	1799	3605	54,038
8	16,453	32,181	1822	3582	54,038
9	16,416	32,219	1859	3544	54,038
10	16,439	32,196	1836	3567	54,038

Each fold has obtained the 1D-CNNs model performance based on five metrics: accuracy, sensitivity, specificity, F-score, and precision (refer to Table 4). Overall, the model has obtained good performance with up to 99.80% accuracy. However, among the tenfold cross validations, the 4th, 8th, and 9th fold achieved 100% in all performance metrics. This means the best folds of 4, 8, and 9 were entirely successful in classifying N and AF. The average accuracy, sensitivity, specificity, F-score, and precision for the two classes (N and AF) in tenfold were 99.8%, 99.8%, 99.8%, 99.77%, and 99.74%, respectively.

**Table 5:** AFibNet performance with tenfold cross validation for two-class

Fold	Classifier performances (%)				
	Accuracy	Sensitivity	Specificity	F1-score	Precision
1	98.22	98.24	98.24	97.98	97.74
2	99.94	99.94	99.94	99.93	99.93
3	99.98	99.98	99.98	99.97	99.97
4	100	100	100	100	100
5	99.96	99.97	99.97	99.95	99.94
6	99.98	99.98	99.98	99.97	99.97
7	99.94	99.94	99.94	99.93	99.93
8	100	100	100	100	100
9	100	100	100	100	100
10	99.98	99.98	99.98	99.97	99.97
Average	99.8	99.8	99.8	99.77	99.74

The optimum ECG sampling rate required for HR analysis to ensure acceptable accuracy of R–R intervals has not yet been determined [49, 50]. Previous studies indicate that a low sampling rate may decrease the accuracy in detection of R–R points, thereby changing the HR parameters [49, 50]. In the present study, the proposed 1-D CNNs model has been tested with various datasets that vary in frequency sampling and lengths of the ECG signal recordings. No duplication exists between the training and testing sets, because they were separated at the beginning of process. Our proposed model therefore overcomes this problem and the ECG signal is still recognized as the N or AF condition.

The performance of the cloud DL approach is listed in Table 6 with the interval of frequency sampling of the ECG devices from 100 to 500 Hz. The proposed 1D-CNNs model has obtained perfect results for N and AF detection with 100% for accuracy, sensitivity, and specificity. The results show good performance without considering the frequency sampling.

**Table 6:** All performance of the AFibNet with several datasets

Dataset	Class	Number of subjects	Performance (%)		
			Accuracy	Sensitivity	Specificity
*Training and validation data*					
The 2017 PhysioNet/CinC challenge	N				
China physiological signal challenge 2018	AF	8232	99.8	99.8	99.8
MIT-BIH atrial fibrillation					
*Unseen data testing*					
ECG long term AF	AF	38	100	100	–
Paroxysmal AF	AF	48	100	100	–
MIT-BIH Arrhythmia	AF	6	100	100	–
AF termination challenge	AF	10	100	100	–
Fantasia	N	24	100	100	–
Indonesian Hospital (ECG 1)	N	42	100	100	100
	AF	3			
Indonesian Hospital (ECG 2)	AF	13	100	100	–
ECG recording from Chapman University and Shaoxing People’s Hospital	N	1646	98.86	98.88	*98.88*
	F	1780			
All unseen data testing	N	1712	98.94	98.97	98.97
	AF	1898			

Training and validation dataset: The sample of data used to fit the and provide an unbiased evaluation of a model fit on the training dataset while tuning model hyperparameters. Unseen data: The unseen data can include data having an attribute not seen by the data set

The methods of AF detection are mainly based on R-R intervals, short-term heart rate variability analysis, and sequential review to verify the presence of P-waves. In this work, the model has been tested using unseen data in order to detect any false positive (FP) and false negative (FN) predictions as a way to clarify the robustness of the technique. As shown in Table 6, when the data are increased (combination of all unseen data), the classification error from the proposed model produce 10 FN and 29 FP which impact the model performances. The performance result achieved of 98.94% accuracy and 98.97% for both sensitivity and specificity. The potential solution to this problem is to remove the noise level in the ECG signal with other filters to maximize the method’s efficiency. In the future, the preprocessing step will be improved in terms of a filter fusion mechanism for noise removal from the ECG recordings.

#### Test case 2: N, AF, and Non-AF classification (three-class)

In the three-class classification, the data distribution produce imbalance class, due to 14,511 records of Non-AF rhythms. In an imbalanced class, a classifier tends to predict the majority of classes effectively. However, the minority class prediction levels are substantially reduced, reducing the model’s reliability levels. Based on Table 7, by using the AFibNet model, even though the number of records is increased with imbalance class among N, AF and Non-AF rhythms, it still produces high performance in accuracy, sensitivity, specificity, precision, and F1-Score, which reveals the ability of the classifier to predict the increase in the minority class. It can be seen from Table 7, by using our AFibNet model, it produces average performance with 96.36% accuracy, 93.65% sensitivity, and 96.92% specificity for three classes. It decreases only 3% accuracy from 11,842 subjects for two classes become 26,349 subjects for three classes.

**Table 7:** Two-class classification performance of the AFibNet with intra-patient mechanism

Fold	Performances (%)				
	Accuracy	Sensitivity	Specifisity	Precision	F1-Score
1	76.29	57.02	79.10	53.19	54.12
2	96.37	94.90	97.28	91.83	93.24
3	98.02	96.64	98.46	95.66	96.14
4	99.08	98.41	99.27	97.97	98.19
5	99.29	98.88	99.46	98.24	98.55
6	98.88	98.13	99.14	97.36	97.73
7	99.34	98.89	99.49	98.41	98.64
8	98.22	97.10	98.46	96.59	96.83
9	99.18	98.48	99.34	98.08	98.28
10	98.91	98.01	99.13	97.39	97.69
Average	96.36	93.65	96.92	92.47	92.94

An in-depth investigation is carried out to ensure the robustness of the selected model from Table 7. For all classes produce 99% accuracy, and for the N class, a perfect sensitivity of 100% is achieved (refer to Table 8). The performance of all validations data produces consistent results for the N, AF, and non-AF conditions, even though the imbalance ratio for the data between N, F and Non-AF. It means our proposed model is ready to be implemented in a real AF detection system. The AFibNet model remains robust in several datasets, and it can be generalized and developed for binary or multi-class classification.

**Table 8:** AFibNet performance for each class

Performance (%)	N	AF	Non-AF
Accuracy	99.89	99.13	99.01
Sensitivity	100	97.92	98.77
Specifisity	99.84	99.33	99.32

### Validating robustness in a cloud server

The convolution process generated a feature map which used as new input data for the next step. The 1D-CNNs model with only simple array operations learns 1D signals with a few hidden layers and neurons. After the nonlinearity process, all characteristics are produced only during each convolution process; this stage never occurs in the pooling process. This process allowed the model to create 64 unique features on the network’s first layer. Due to its ability to avoid the vanishing gradient in the training process, we use ReLU as a nonlinearity function. The product of the layer of convolution is called the map of the function. We added the pooling layer after the second convolution layer. The purpose of this layer is to reduce the size of the feature map to lower the complexity. The max pooling layer is used since it can extract the essential features from the feature map. Two hidden layers in the fully connected part are created, each with 1000 nodes, while the output layer defines a sigmoid function to classify the ECG data.

At present, the computing scene has become very diverse regarding computing platforms. A number of unique accelerators have been created, in addition to the wide variety of GPUs available for CNNs computations. The size ranges from small low-power systems to computing on the warehouse-scale [51]. Meanwhile, the CPU development [13] has continued and many CPUs offer acceleration for CNNs computations. The same diversity applies to runtime systems [43]. The computational specification and performance of CNNs for AF detection are not yet well understood. In order to calculate the computational complexity of a CNN, the original implementation of the CNNs algorithm is needed. In the present study, the Keras library is utilized to implement the CNNs algorithm based on parallel processing for the training process so that it is unable to present the exact complexity of the CNNs algorithm. However, the weight parameters for every layer are calculated to predict the computational consumption. Our proposed 1D-CNNs model has 13 convolution layers with 5 polling layers, and the consecutive layers produce about 45, 846, 329 weight parameters. All parameters are depicted in Table 9. However, the only operation with a significant cost is a sequence of 1D convolutions which are simply linear weighted sums of two 1D arrays. Such a linear operation during the forward and backward operations can effectively be executed in parallel. It means although the parameters are a lot, it does not increase the computation time and resources.

The 1D-CNNs model is analyzed to determine the speed of the processing time needed to predict the ECG signal from the raw data and arrive at a decision in the cloud system. The whole process is divided into four stages: read data, denoising, load model, and inference. The computational consumption mostly involves the load model and inference stages. While the number of weight parameters is high, the execution time is quite negligible. For each relation, only scalar weight multiplication and addition are performed.

**Table 9:** The number of parameters produce based on 1D-CNNs architecture to show the computational complexity

Layer name	Output shape	Parameters
Convolution 1	(None, 2698, 64)	256
Convolution 2	(None, 2696, 64)	12,352
Maxpooling 1	(None, 1348, 64)	0
Convolution 3	(None, 1346, 128)	24,704
Convolution 4	(None, 1344, 128)	49,280
Maxpooling 2	(None, 672, 128)	0
Convolution 5	(None, 670, 256)	98,560
Convolution 6	(None, 668, 256)	196,864
Convolution 7	(None, 666, 256)	196,864
Maxpooling 3	(None, 333, 256)	0
Convolution 8	(None, 331, 512)	393,728
Convolution 9	(None, 329, 512)	786,944
Convolution 10	(None, 327, 512)	786,944
Maxpooling 4	(None, 163, 512)	0
Convolution 11	(None, 161, 512)	786,944
Convolution 12	(None, 159, 512)	786,944
Convolution 13	(None, 157, 512)	786,944
Maxpooling 5	(None, 78, 512)	0
Flatten	(None, 39936)	0
Dense	(None, 1000)	39,936,000
Dense	(None, 1000)	1,001,000
Class	(None, 1)	1001
Total of parameters		45, 846, 329

This paper investigates the computational behavior and performance of AF detection from short-term ECG signals using 1D-CNNs. Table 10 lists the four computer specifications in the cloud in this work. The test was conducted to predict the AF condition in unknown data from short-term ECG signals using several datasets. Using the 4th computer specification (refer to Table 10) and utilizing the GPU memory, a prediction of an AF condition takes 0.02 s. This means that high specifications for the CPU and GPU result in faster processing in the cloud system.

The throughput time, inference time, and memory consumption are also calculated in this work. The throughput is the number of instances that can be transmitted in one second on the network. We would like to process a single instance in as many instances as possible in parallel to reach the optimum throughput. A good rule of thumb is to hit the memory limit of the GPU for the specified data form to find the best network. This size depends on the type of hardware and the network size. As shown in the results, the entire AF classification phase can still be processed with good performance, and the 1D-CNNs provide low computational complexities at acceptably low cost with low power hardware.

**Table 10:** The sample of CPU and GPU process as a cloud server

Specification	CPU	GPU	Testing (s)
1	CPU1: 4 Core, 8 thread, @2.8 GHz	–	0.30
	Memory: 16 GG, Disk: 1000 Gb		
2	CPU1: 4 Core, 8 thread, @2.8 GHz	GPU1: GTX	0.18
	Memory: 16 Gb, Disk: 1000 Gb	1050 Ti, 4Gb	
3	CPU2: 8 Core, 16 thread, @3.6 GHz	–	0.14
	Memory: 32 Gb, Disk: 1000 Gb		
4	CPU2: 8 Core, 16 thread, @3.6 GHz	GPU2: RTX	0.02
	Memory: 32 Gb, Disk: 1000 Gb	2080 Ti, 11Gb	

**Fig. 4:**
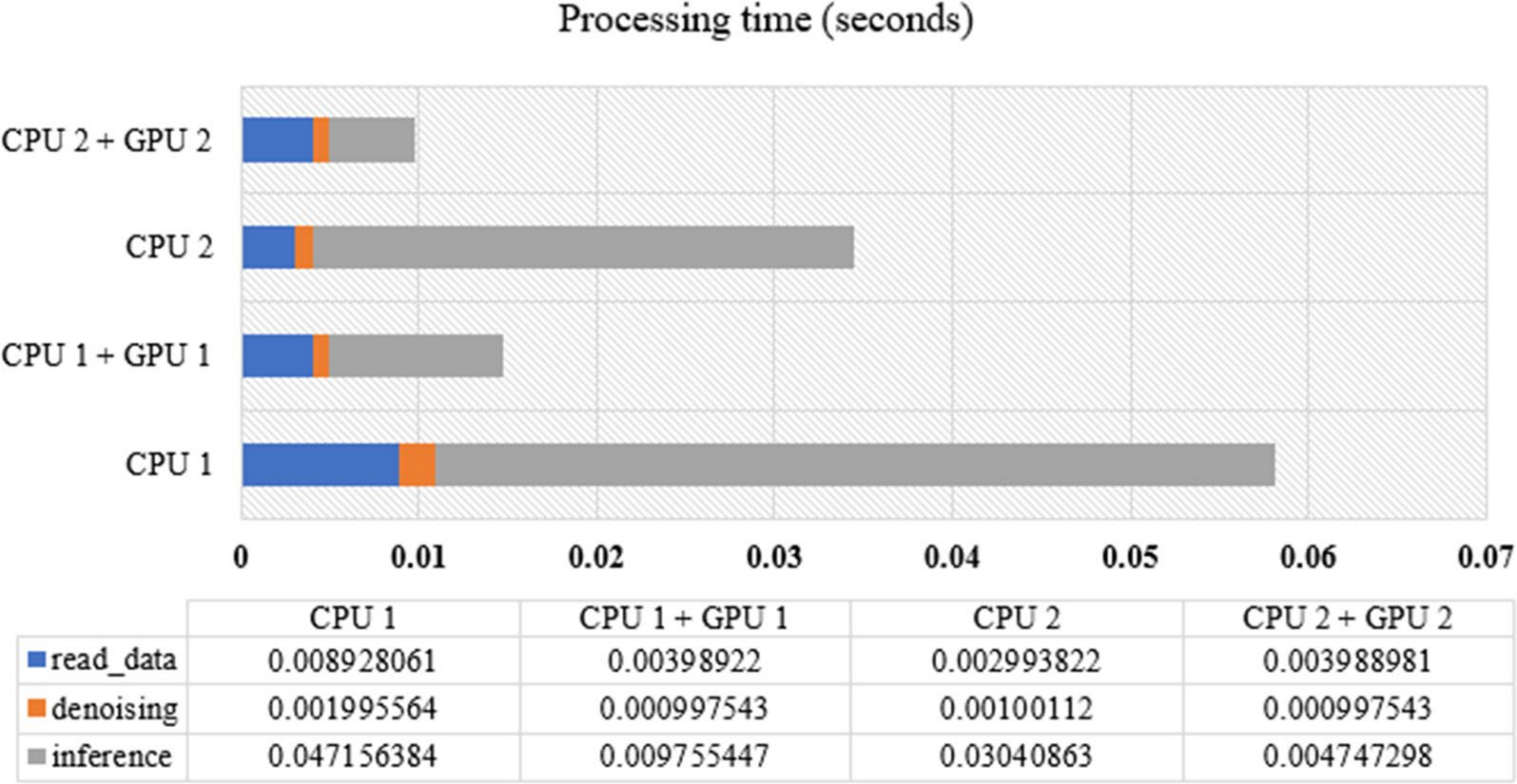
Processing time of 1D-CNNs in four server specification

Figure 4 illustrates the total time for the model to inference the input data. This process consists of three main processes: data reading, denoising, and inferencing. The processes of reading the data and denoising show no significant time differences among the four servers. However, when entering the inference step, servers equipped with GPUs have faster processing times compared to servers without GPUs. The time difference is quite striking due to the ability of the GPU to parallelize the process during the inference step. Overall, servers with CPU 2 + GPU 2 specifications have the fastest processing times compared to the others.

**Fig. 5:**
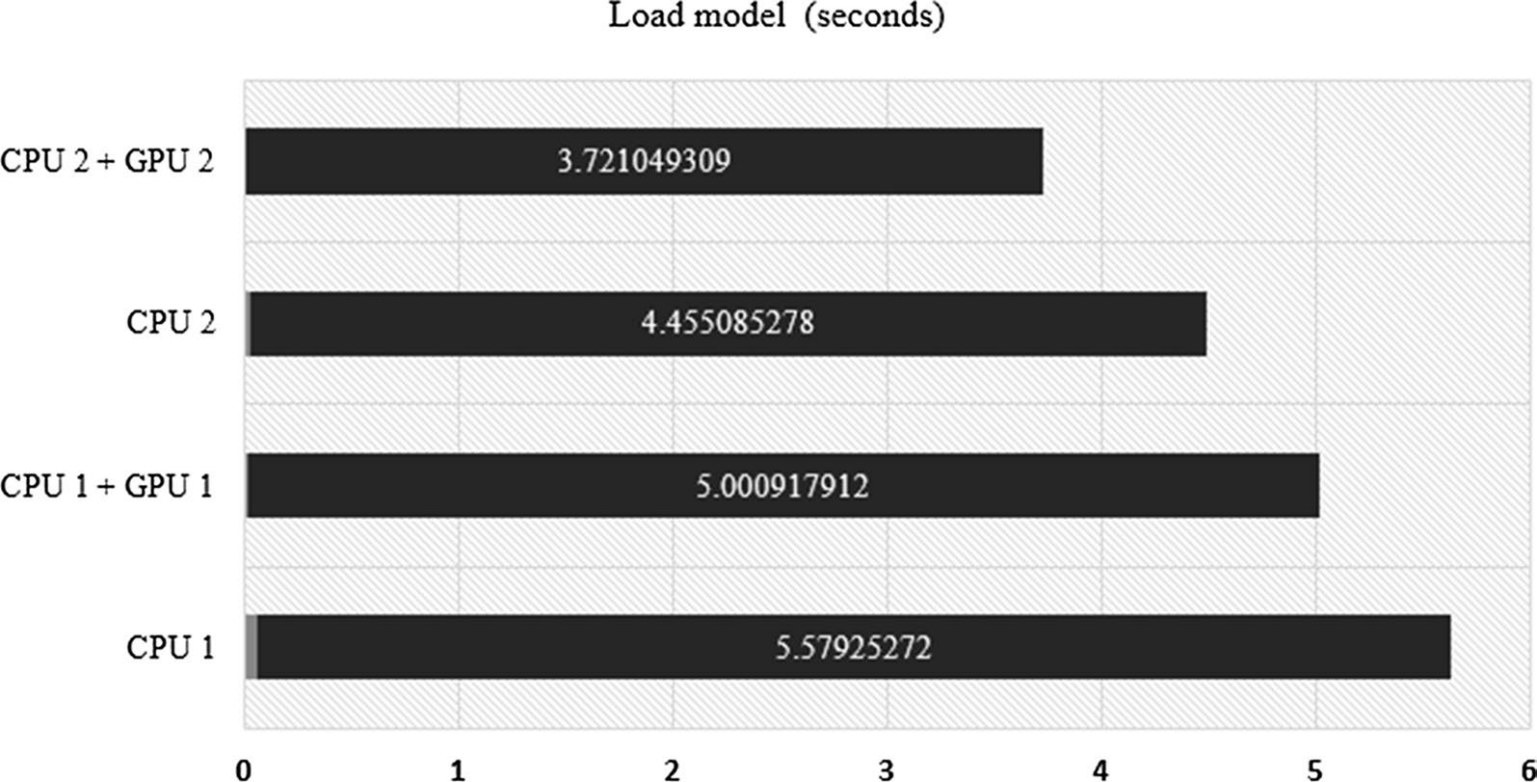
Processing time of load model in cloud system

Another aspect that is also quite important in analyzing the robustness of a cloud server is the processing time for loading the model. Although this process is only done once (when the model is deployed to the server), this step is also quite important because the size of the DL model is relatively large and the processing time also takes time. In this paper, the size of DL model is more than 500 Mb. As shown in Fig. 5, the server with CPU 2 + GPU 2 has the fastest time for loading the deep learning model, at 3.7 s. This is because the server has the largest GPU memory compared to the others, so the process of model reading is faster.

**Fig. 6:**
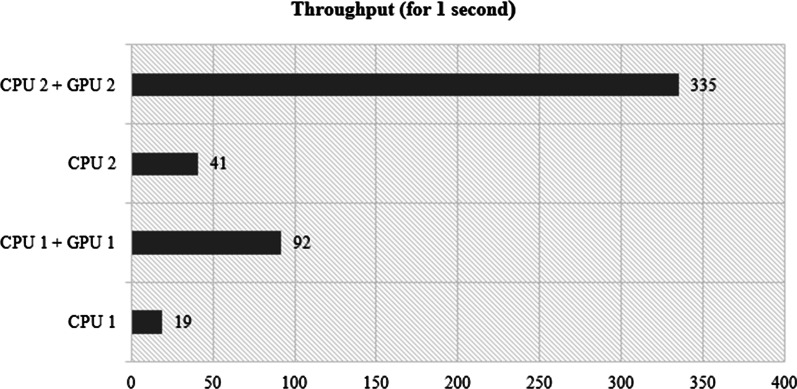
Throughput

The last aspect tested is the throughput time of the server. In this test, the reliability of the four server specifications in serving inference requests is assessed. The length of time used in testing the throughput time is one second. Figure 6 shows that servers with CPU 2 + GPU 2 have the largest number of services, at 335. This is directly proportional to the total inference time, which only takes 0.0079 s to predict input data (Fig. 4). For single ECG signal prediction, the dominating delay is the 1D-CNNs model loading and neural network setup, with the actual inference being comparatively fast on all frameworks and with both models. With the advance of GPU technology, our DL model can approximate a very complicated learning function with a reasonable training time. 1D-CNNs can make predictions directly from raw data; hence the effectiveness of the learning process is increased when large datasets are available.

### Benchmarking over other DL algorithms with the cloud system

This study achieved 100% accuracy for unseen data testing of two-class case with different frequency samplings and datasets (refer to Table 5). The proposed 1D-CNNs model obtained robust performance under several conditions. For clinical use, the AI-aided ECG AF diagnostic method we developed appears to be sufficiently accurate. For major general hospitals, it may help to minimize misdiagnosis, thereby saving labor costs. This study has also solved the ECG signal problem regarding unequal signal lengths, frequency sampling, and imbalanced data. This study has also compared the previous studies that used both limited and large ECG datasets.

Some previous studies have explored the performance of the cloud for AF detection based on deep learning approaches, such as autoencoders, CNNs, and LSTMs (refer to Table 11). For example, Faust et al. [4] detected episodes of AF using heart rate signals and RNNs with a LSTM model. The RNNs with the LSTM model provided the intelligence needed for state-of-the-art IoT-based diagnosis support systems. They trained and tested with labeled HR signal data from 20 subjects sourced from PhysioNet Atrial Fibrillation Database (AFDB) and blindfolded validation, using the data from 3 subjects from AFDB and 82 subjects sourced from the Long-Term AF Database. Both performances of the dataset achieved 99.77% and 94% accuracy. Hong et al. [3] introduced their work on building, training, and serving an out-of-the-box cloud deep learning service they called CardioLearn for cardiac disease detection from ECGs. They used the ECG data with two forms of input: single lead and 12-lead. They tested CNN-RNN as the proposed approach on the 2018 China Physiological Signal Challenge dataset and achieved 98.57% and 97.89% receiver operating characteristics (ROC) and the area under the ROC (ROC-AUC) scores for single lead and 12-lead data. They also designed a portable smart hardware device, along with an interactive mobile program, to demonstrate its practical use. Zhang et al. [52] established the Cardiovascular Disease Whole Process Management Platform for automated detection and classification of ECG signals. They obtained 98.27% accuracy for recognition of 18 classes of heart rhythms based on a CNNs model. Their proposed model also achieved 99.95% sensitivity for AF detection and 85.49% accuracy and 88.52% sensitivity for normal cases. Yildirim et al. [53] proposed an effective deep neural networks (DNNs) to detect different rhythm classes from ECG databases. With the 1,780 AF and 1,825 normal cases, the class-based performance achieved an average 97.91% accuracy, 96.52% sensitivity, and 98.31% specificity from Lead II-inputs for AF and normal sinus detection.

In the present study, we have proposed a one-dimensional CNNs for AF detection. We have experimented the testing (unseen data) with and without ECG recordings from Chapman University and Shaoxing People’s Hospital. Without the database, total records of 8,416 were achieved with 100% accuracy, sensitivity, and specificity. We then added more data to 11,842 subjects for two-class cases and obtained 98.94% accuracy, 98.97% sensitivity, and 98.97% specificity. When we have applied for three-class case, with total of 26,349 data, the performance achieved 96.36 accuracy, 93.65% sensitivity, and 96.92% specificity. Although the performance results were decreased, the proposed model was still reliable for AF detection.

**Table 11:** Benchmarking with other DL for AF detection

Authors	Method	Total subject	Acc. (%)	Sens. (%)	Spec.	ROC-AUC Score (%)
Faust et al. [4]	RNNs-LSTM	102	98.51	–	–	–
Hong et al. [51]	CNNs-RNNs	± 20,000	–	–	–	98.57
Zhang et al. [52]	CNNs	177,941	91.88	94.23	–	–
Yildirim et al. [53]	DNNs	3605	97.91	96.52	98.31	–
Proposed model	1D-CNNs	8416	100	100	100	–
		11,842	98.94	98.97	98.97	–
		26,349	96.36	93.65	96.92	-

*Acc* Accuracy, *Sens* Sensitivity, *Spec* Specificity

In summary, we demonstrate that an end-to-end approach using 1D-CNNs will classify AF from single-lead ECGs from a wide variety of separate instruments with a diagnostic efficiency close to that of cardiologists. If verified in clinical settings, this methods has the potential to enhance the precision, performance, and scalability of ECG interpretation. However, our generalization model of a 1D-CNNs also has some limitations, which can be summarized as follows:

1. Our method is validated only for N, AF and Non-AF detection, whereas a wide variety of different arrhythmias detected from single-lead ECGs need to be classified in the future and researched in depth to confirm a high diagnostic output close to that of cardiologists;
2. The proposed single learning method will be tailored to the target application prior to clinical application, which could entail additional pre- or post-processing steps;
3. Our DL-cloud architecture, which was not focused on the calculations of actual workloads and real computing platforms, was only available for neural network inference in terms of software frameworks and hardware acceleration.

## Conclusion

AF has a high risk of severe health consequences, including death and stroke. Therefore, continuous AF monitoring could have a beneficial clinical impact by allowing the identification of AF in patients with post-ablation chronic AF or pharmacological cardioversion, for example. Our study is the first comprehensive demonstration of a DL approach to perform classification across a broad range of the most common and important ECG rhythm diagnoses with large datasets. We highlight the differences in the length of the ECG recording, the frequency sampling, and the data acquisition devices. This highlights the ability of our end-to-end 1D-CNNs-based approach to generalize the cloud deep-learning approach to a new set of AF rhythm labels on a number of datasets.

The approach exhibited rapid adoption that provides a chance for highly scalable AF detection. In the current study, we have trained and validated varied data with different frequency sampling. We also used unseen data from public and Indonesian hospital datasets to measure the robustness of proposed model. All ECG recordings were segmented into 2700 samples, which can present up to two R-R intervals. The 1D-CNNs model with 13 convolutions and 5 max-pooling layers reached the two-class classification performance of 99.80% accuracy, sensitivity, and specificity in the training and validation data. The unseen data from 3,610 records used as blindfold validation revealed that the model achieved 98.94% accuracy, 98.97% sensitivity, and 98.97% specificity. Whereas three-class classification performance produce, 96.36% accuracy, 93.65% sensitivity, and 96.92% specificity, respectively.

We also tested the scalability of the proposed model for different server specifications, such as a cloud server. The AFibNet was capable of generating an AF prediction quickly, indicating that our DL-based 1D-CNNs model has outstanding performance results. For functional diagnostic assistance, this concept is important since using information gained over a limited training period is precisely what a cardiologist does. In the future, the recommended model could be used to better classify AF patients early on, so that they can be managed to avoid stroke.

## Data Availability

All the data considered for this study is available at PhysioNet, https://physionet.org/.
